# Analysis of the different characteristics between omental preadipocytes and differentiated white adipocytes using bioinformatics methods

**DOI:** 10.1080/21623945.2022.2063471

**Published:** 2022-05-01

**Authors:** Xinyu Yang, Lu Li, Canming Xu, Meichen Pi, Changhua Wang, Yemin Zhang

**Affiliations:** a Department of Pathology & Pathophysiology, Wuhan University Taikang Medical School (School of Basic Medical Sciences), Wuhan Hubei China; b Hubei Provincial Key Laboratory of Developmentally Originated Disease, Wuhan Hubei China; c Demonstration Center for Experimental Basic Medicine Education of Wuhan University Taikang Medical School (School of Basic Medical Sciences), Wuhan Hubei China

**Keywords:** Differentiated white adipocyte, preadipocytes, different characteristic, bioinformatics, GSAE

## Abstract

Obesity is emerging as an epidemiological issue, being associated with the onset and progress of various metabolism-related disorders. Obesity is characterized by the white adipose expansion, which encounters white adipocyte hypertrophy and hyperplasia. White adipocyte hyperplasia is defined as adipogenesis with the increase in the number of the white adipocytes from the preadipocytes. Adipogenesis contributes to distributing excess triglycerides among the smaller newly formed adipocytes, reducing the number of hypertrophic adipocytes and secreting anti-inflammatory factor. Therefore, adipogenesis is emerging as a new therapeutic target for the treatment of obesity. In the present study, for a better understanding of the contribution of the alteration of the omental differentiated white adipocytes to the systemic metabolic disorders, we downloaded the mRNA expression profiles from GEO database GSE1657, 328 differentially expressed genes (DEGs) were screened between the undifferentiated preadipocytes (UNDIF) and omental differentiated white adipocytes (DIF). The contributions of the upregulated and downregulated DEGs to the system were performed via the Gene Ontology (GO) analysis, the Kyoto Encyclopedia of Genes and Genomes (KEGG) pathway analysis and Protein–Protein Interaction (PPI) network, respectively. The potential contribution of the whole altered genes in the differentiated white adipocytes was explored with the performance of Gene Set Enrichment Analysis (GSEA), especially on the GO analysis, KEGG analysis, hallmark analysis, oncogenic analysis and related miRNA analysis. The output of the current study will shed light on the new targets for the treatment of obesity and obesity-related disorders.

## Introduction

Obesity is emerging as an epidemiological issue, being associated with the onset and progress of various metabolism-related disorders such as insulin resistance, type 2 diabetes, cancers, diabetic cardiomyopathy and so on [1–3]. Obesity is due to the expansion of white adipocyte, which is achieved not only by hypertrophy, which is defined as the increase in the size of the white adipocyte storing excess energy in the form of triglycerides, as the lipid-droplet white adipocyte, but also by hyperplasia, as adipogenesis with the increase in the number of the white adipocytes from the preadipocytes [4].

With the white adipose expansion, the white adipose enlarges the volume, furthermore, there assumes a paradoxical state, on the one hand, lipid-droplet white adipocytes encounter hypoxia, the recruitment of inflammatory factors, leading to ectopic fat deposition in liver and heart, systemic glucose intolerance and insulin resistance [5,6]. However, on the other hand, with the increase in the number of white adipocytes, adipogenesis distributes excess triglycerides among the smaller newly formed adipocytes, reducing the number of hypertrophic adipocytes, secreting anti-inflammatory factors [7,8], various adipokines, mRNAs, noncoding RNAs to improve the internal metabolism and other tissue metabolisms via exocrine function and the exosomes, enlarging the area of contact with blood vessel to alleviate hypoxia, which are followed by the improved white adipocytes and the systemic insulin resistance [9,10]. Therefore, a better understanding of the alteration of the white adipocytes with the adipogenesis of preadipocytes would empower us to pursue the new strategy for the treatment of obesity and the metabolic related disorders.

In the present study, to better understand the contribution of the alteration of the white adipocytes differentiation to the systemic metabolic disorders, we downloaded the mRNA expression profiles from Gene Expression Omnibus (GEO, http://www. ncbi.nlm.nih.gov/geo/), an international public repository providing freely high-throughput microarray and relevant functional genomic data sets [11]. The samples were derived from the human omental preadipocytes collected from the subjects undergoing the gastric bypass surgery for the management of obesity, which were treated with a differentiation medium or not. With the performance of limma package in R software, 328 differentially expressed genes (DEGs) were screened between the undifferentiated preadipocytes (UNDIF) and the differentiated white adipocytes (DIF) groups [12]. The contributions of the upregulated and downregulated DEGs to the system were analysed via the Gene Ontology (GO) analysis, the Kyoto Encyclopedia of Genes and Genomes (KEGG) pathway analysis and Protein–Protein Interaction (PPI) network, respectively. The potential contribution of the whole altered genes in the DIF group was explored with the performance of Gene Set Enrichment Analysis (GSEA), especially on the KEGG analysis, hallmark analysis, oncogenic analysis and the altered genes related miRNA analysis. The outputs of the present study will shed light on the new targets for the treatment of obesity and the obesity-related disorders.

## 2 Materials and methods

### 2.1 Omental preadipocytes obtention and differentiation to mature adipocytes

The omental preadipocytes were resected from the subjects [3 female at the age under 40 yr, median body mass index (BMI) 47.8, range 44.4 to 51.1 kg/m^2^, fasting glucose 105 + 6.48%mg], undergoing the gastric bypass surgery for the management of obesity. The omental fat tissue was collected in Hank’s balanced salt solution with bicarbonate, penicillin, and gentamicin, followed by the omental preadipocytes were detached with the method described previously in the GEO database GSE1657 (http://www.ncbi.nlm.nih.gov/geo). After the confluence of omental preadipocytes, for differentiation, the preadipocytes were treated with cocktail method with the plating medium (without serum) enriched with 100 nM dexamethasone, 500 nM human insulin, 200 pM triiodothyronine, 0.5 nM rosiglitazone, antibiotics, and 540 nM methylisobutylxanthine for 2 days, followed with the plating medium (without serum) enriched with 100 nM dexamethasone, 200 pM triiodothyronine, antibiotics, and 540 M methylisobutylxanthine for another 26 days. For the following 2 days, differentiated adipocytes were cultured in a plating medium without serum. In addition, undifferentiated preadipocytes were cultured with the plating medium. The description of the whole process was derived from the GEO database GSE1657 (http://www.ncbi.nlm.nih.gov/geo).

### 2.2 Microarray data archives

The expression profiles by an array of GSE1657 were retrieved from GEO database. The samples were derived from the human omental preadipocytes collected from the subjects undergoing the gastric bypass surgery for the management of obesity, which were treated with differentiation medium or not. The expression profiling of the database was based on the GPL96 (Affymetrix Human Genome U133A Array) platform. A series of matrix files and data table header descriptions of the database were downloaded from the GEO database GSE1657 for the subsequent analysis.

### 2.2 Microarray data and DEGs identification

Following the databases annotated by the performance of Perl script, the limma package in R software (version 3.6.3) (University of California, Berkeley, CA) was applied to screen the DEGs with the threshold criterion of adjusted p < 0.05 and |log_2_ FC|; (foldchange) >1 between the UNDIF and DIF white adipocytes group [12]. The pheatmap package in R software was subsequently performed to plot the heatmap of DEGs [13].

### 2.3 PPI network creation and hub gene identification

The PPI network of the DEGs was constructed by Search Tool for the Retrieval of Interacting Genes (STRING11.5; https://string-db.org/) with a combined score >0.7 as the cut-off point [14]; then, the network was visualized with the performance of Cytoscape software (Cytoscape, 3.7.1) [15]. The significant modules in the PPI network were identified by the molecular complex detection (MCODE 1.5.1), one plug-in of Cytoscape software [16]. The parameters of DEGs clustering and scoring were set as follows: MCODE score ≥4, degree cut-off = 2, node score cut-off = 0.2, max depth = 100, and k-score = 2.

### 2.4 GO and KEGG pathway enrichment analyses

GO is a commonly used bioinformatic tool that provides the comprehensive information on the gene function of individual genomic products based on the defined features. GO analysis and KEGG pathway analysis of the DEGs were performed via The Database for Annotation, Visualization, and Integrated Discovery (DAVID 6.8, http://david.ncifcrf.gov) [17]. The GO analysis consists of biological processes (BP), and cellular components (CC), molecular functions (MF). KEGG is a database resource for understanding high-level biological functions and utilities. Gene count >2 and p < 0.05 were set as the threshold.

### 2.5 GSEA analyses of all altered genes

GSEA is a promising and widely used software package, which derives gene sets to determine the different biological functions of the whole genes between the UNDIF and DIF omental white adipocytes groups. The potential contribution of the whole altered genes in the white adipocytes were explored by the GSEA software (version 4.1.0) [18], especially on the GO analysis, KEGG analysis, hallmark analysis, oncogenic analysis and the altered genes-related miRNA analysis.

### 2.6 Statistical analyses

The statistical analysis of the DEGs was done in the R software with the threshold criterion of adjusted p < 0.05 and |Log_2_ FC|; (foldchange) >1 between the UNDIF and DIF omental white adipocytes groups. The p-values in the GSEA analysis were analysed with the GSEA software (version 4.1.0). During the analysis of GO and KEGG, gene count >2 and p < 0.05 were set as the threshold.

## Results

### 3.1 Identification of DEGs related to omental WAT differentiation

**Figure 1.**
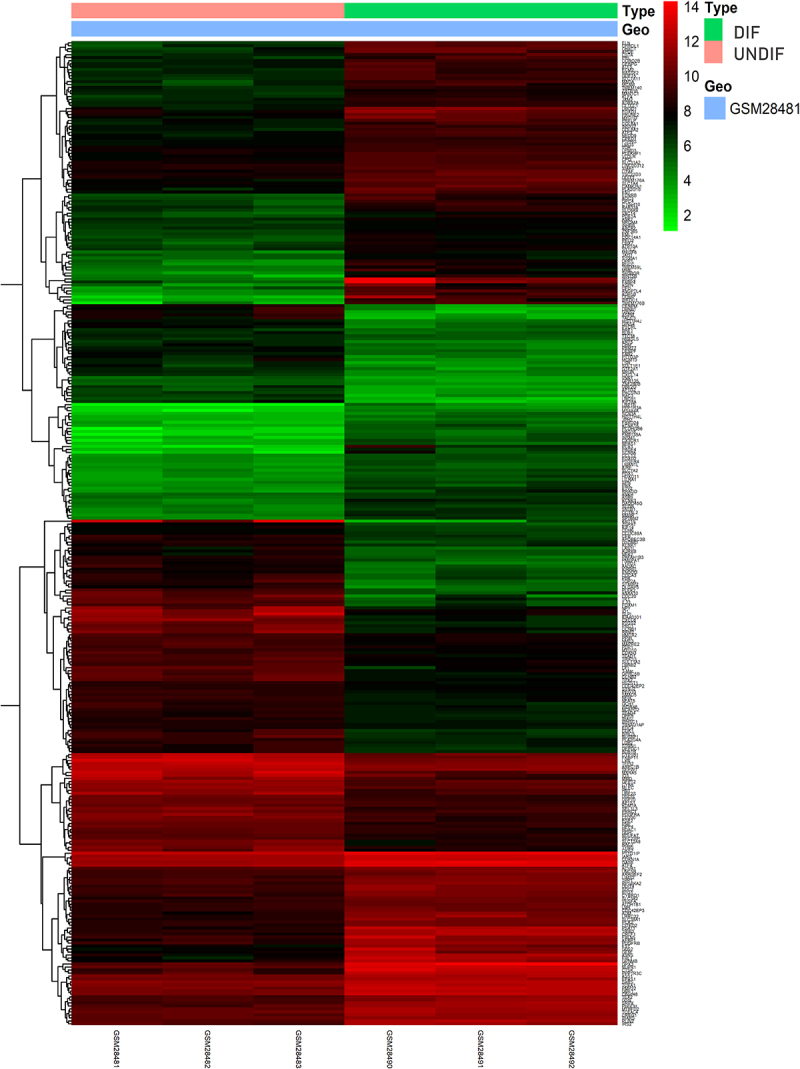
Heatmap of 328 DEGs screened by limma package in R software. Red areas represent upregulated genes and green areas represent downregulated genes in the DIF and UNDIF group. DEG: differentially expressed gene; DIF: differentiated white adipocyte; UNDIF: undifferentiated preadipocytes.

**Figure 2.**
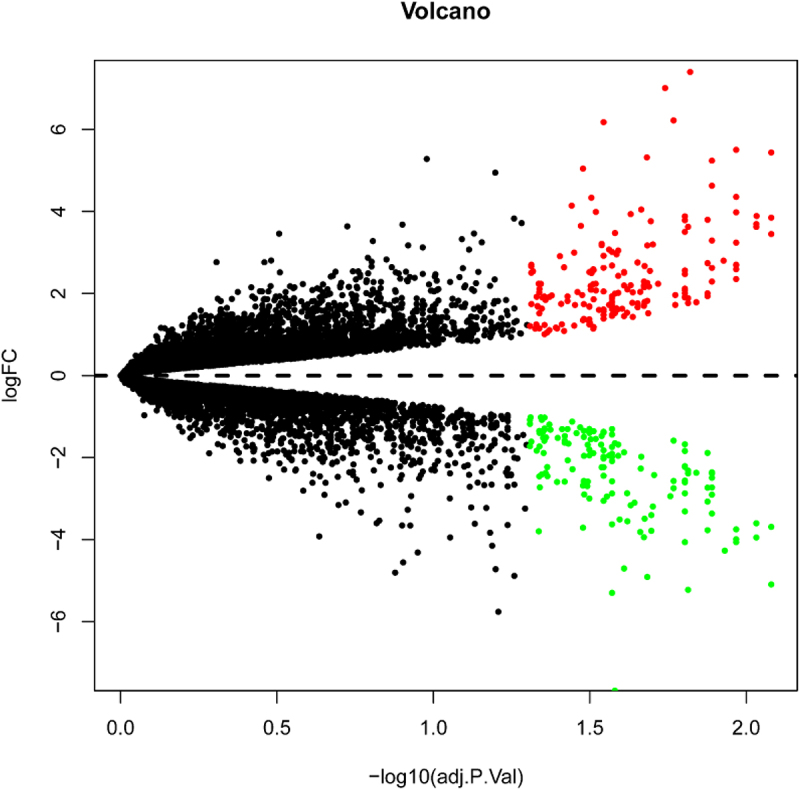
Volcano plot analysis identifies DEGs. Red dots represent 185 upregulated genes and green dots represent 143 downregulated genes from the DIF and UNDIF group.

To identify the DEGs between the UNDIF and DIF groups, we retrieved the relevant microarray expression profiles of GSE1657 from GEO database. After consolidation and normalization of the microarray data, 328 DEGs between UNDIF and DIF omental white adipocytes groups were screened by the limma package (|Log_2_FC| >1, adjusted p < 0.05), as shown in the heatmap (Figure 1). Among them, 185 genes were upregulated and 143 genes were downregulated (Figure 2, Table S1).

### 3.2 PPI network analysis

**Figure 3.**
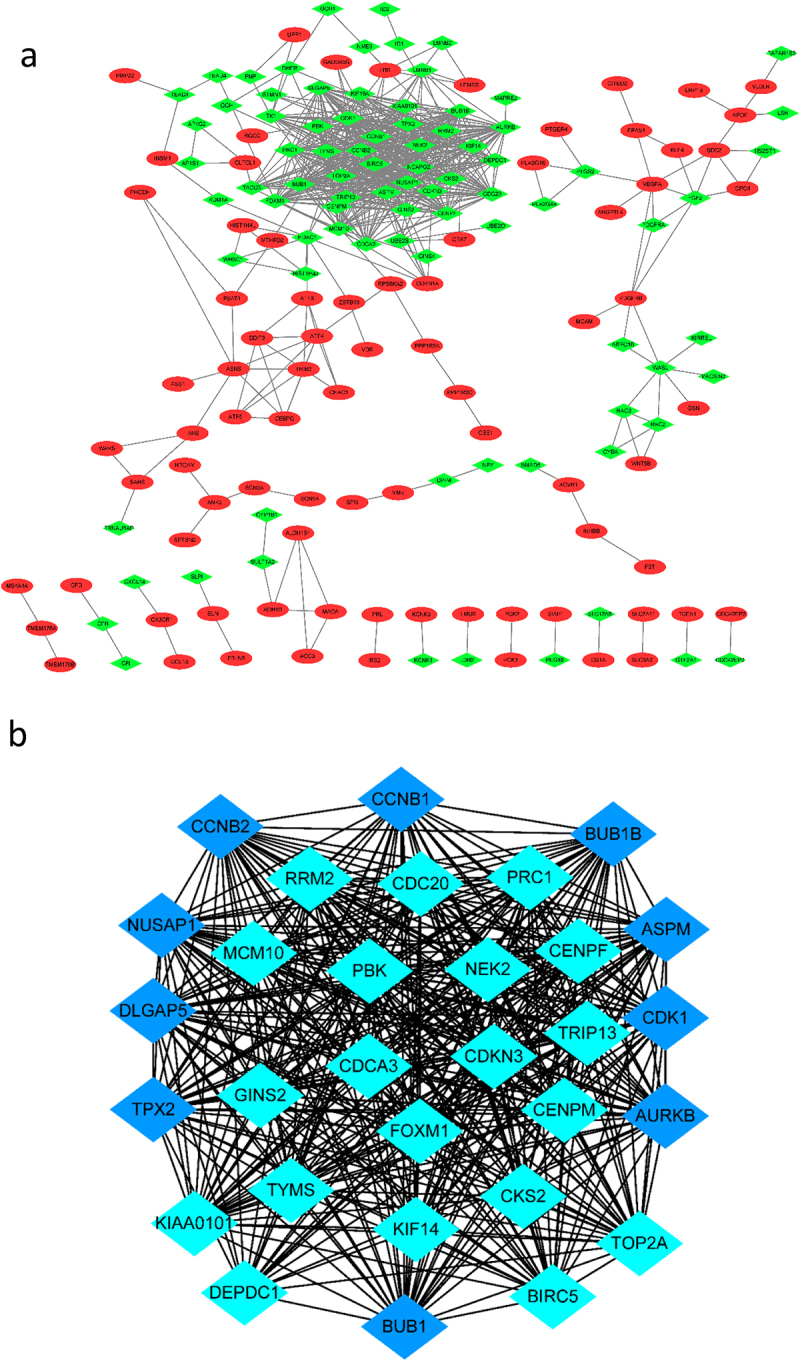
The PPI network of DGEs and the most significant modules of DEGs. (a) The PPI network was analysed by String software. There were 325 nodes and 655 edged in the PPI network. Red represents upregulated genes; green represents downregulated genes. (b) The most significant module identified by MCODE (score = 27.863) Dark blue represents the top 10 genes with the most neighbours and expanded nodes. DEG: differentially expressed gene; PPI: protein–protein interaction.

To identify the most significant clusters of the DEGs, the PPI network of the DEGs was constituted by STRING (11.5). As shown in Figure 3a, there were 325 nodes and 655 edged in the PPI network. The most significant modules (score = 27.863) were recognized by MCODE, a plugin of Cytoscape. The top 10 genes with the most neighbours and expanded nodes included CCNB1, BUB1B, CNNB2, NUSAP1, DLGAP5, TPX2, ASPM, CDK1, AURKB and BUBI (Figure 3b, Table 1).

**Table 1.** Top 10 genes with the most neighbours and expanded nodes in Cytoscape

Gene symbol	Description	logFC
ASPM	asp (abnormal spindle) homolog, microcephaly associated (Drosophila)	−5.09168
DLGAP5	discs, large (Drosophila) homolog-associated protein 5	−3.55184
CCNB1	cyclin B1	−3.19147
NUSAP1	nucleolar and spindle associated protein 1	−2.94063
CCNB2	cyclin B2	−2.73109
CDK1	cyclin-dependent kinase 1	−2.56781
AURKB	aurora kinase B	−2.55832
TPX2	TPX2, microtubule-associated	−2.42251
BUB1B	BUB1 mitotic checkpoint serine/threonine kinase B	−1.89074
BUB1	BUB1 mitotic checkpoint serine/threonine kinase	−1.55189

### 3.3 GO enrichment analysis of the DEGs

**Figure 4.**
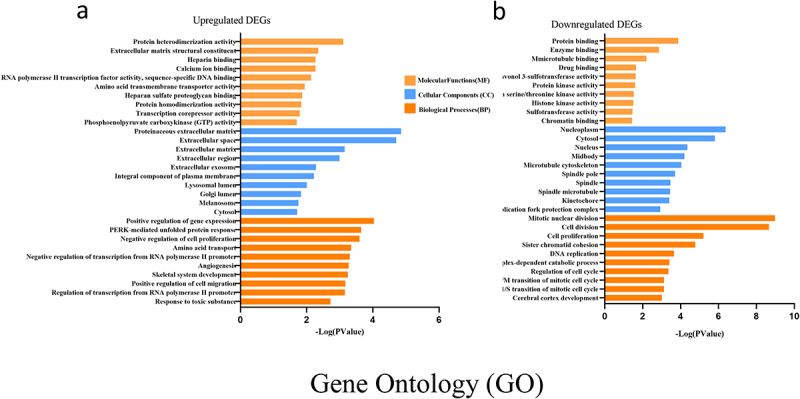
GO enrichment result of upregulated and downregulated DEGs respectively. Abscissa represents -LOG (pValue), and ordinate represents BP, CC, and MF terms. GO: Gene Ontology. BP: biological processes, CC: cellular components, MF molecular functions (MF).

To determine the biological features of the DEGs, GO analysis of the upregulated DEGs and the downregulated DEGs was accomplished, respectively, by DAVID online tools. GO analysis includes BP (biological processes) analysis, CC (cellular components) analysis and MF (molecular functions) analysis. In the present study, the BP analysis revealed that the upregulated DEGs were major enriched in positive regulation of gene expression, PERK-mediated unfolded protein response and negative regulation of cell proliferation (Figure 4a). The CC analysis showed that the upregulated DEGs were enriched in proteinaceous extracellular matrix, extracellular space and extracellular matrix (Figure 4a). The changes in MF of the upregulated DEGs were significantly enriched in protein heterodimerization activity, extracellular matrix structural constituent and heparin binding (Figure 4a).

While the BP analysis of the downregulated DEGs was major enriched in mitotic nuclear division, cell division and cell proliferation (Figure 4b). The CC analysis of the downregulated DEGs was enriched in nucleoplasm, cytosol and nucleus (Figure 4b). The changes in MF of the downregulated DEGs were significantly enriched in protein binding, enzyme binding and microtubule binding (Figure 4b) (p < 05).

### 3.4 KEGG enrichment analysis of the DEGs

**Figure 5.**
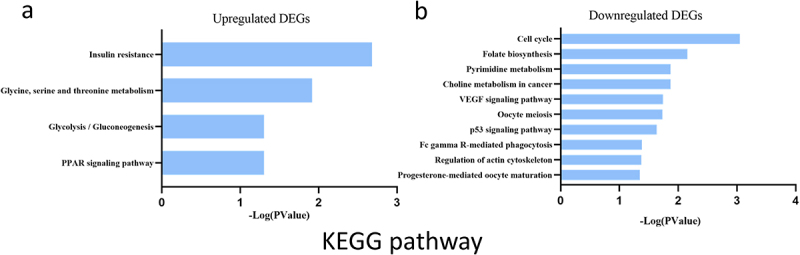
KEGG enrichment result of upregulated and downregulated DEGs respectively. DEGs. Abscissa represents -Log (pValue), and ordinate represents KEGG terms. KEGG: Kyoto Encyclopedia of Genes and Genomes.

To explore the potential mechanism of these DEGs, KEGG pathway analysis was performed using DAVID online tools. The results of KEGG analysis revealed that the upregulated DEGs were mainly involved in insulin resistance, glycine/serine and threonine metabolism, glucolysis/gluconeogensis and PPAR signalling pathway (Figure 5a). However, the results of KEGG analysis revealed that the downregulated DEGs were mainly involved in cell cycle, folate biosynthesis and pyrimidine metabolism (Figure 5b).

### 3.5 GSEA enrichment analysis of the whole altered genes

**Figure 6.**
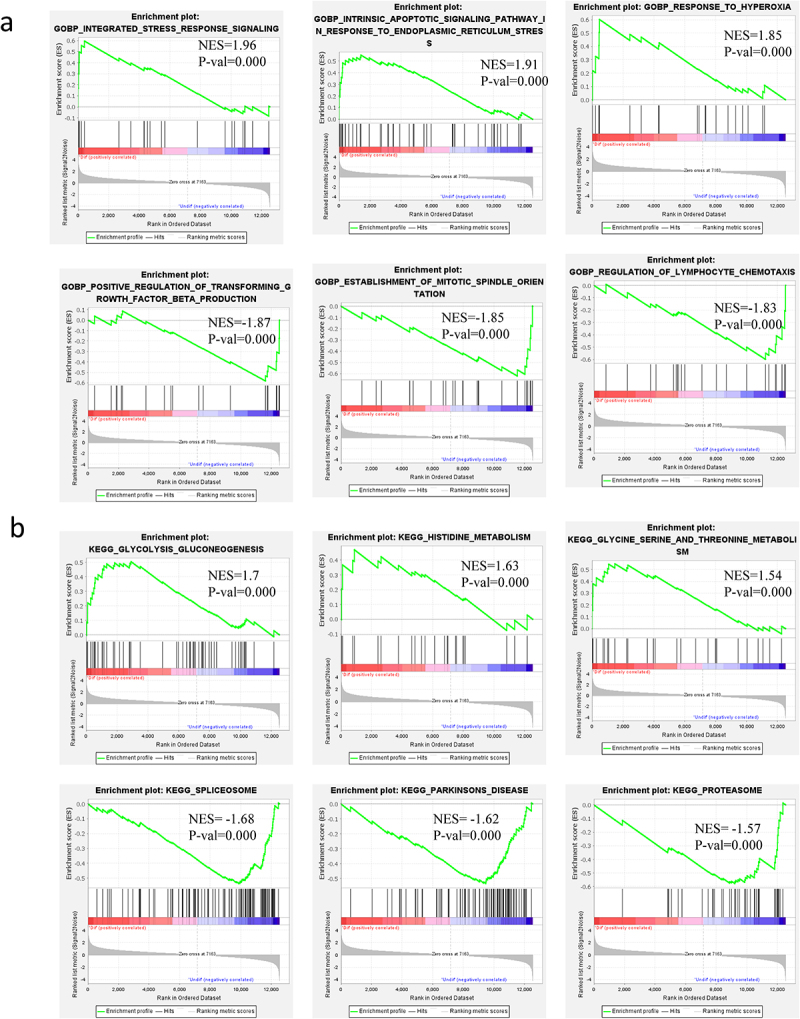
Gene sets enrichment in GSEA analysis of all altered genes on GOBP, KEGG. a: BP of GO analysis of all altered genes in GSEA. b: KEGG analysis of all altered genes in GSEA.

To identify gene sets with a statistically significant difference between the UNDIF and DIF groups, GSEA was performed, which would show the most enriched gene sets of all altered genes between the two groups. The potential contribution of the whole altered genes to the differentiated white adipocytes was explored via GSEA software (version 4.1.0), especially on the BP of GO analysis, KEGG analysis, hallmark analysis, oncogenic analysis, and the altered genes related miRNA analysis. The information of gene sets enrichment on GOBP and KEGG is exhibited in Figure 6a, 6b, Table 2). The information of gene sets enrichment on hallmark analysis, oncogenic analysis and the altered genes-related miRNA is exhibited in Figure Sa, Sb, Sc and Table S2.

**Table 2.** Gene sets enrichment in GSEA analysis on GOBP and KEGG

Type of GSEA	Correlated with the differentiated white adipocytes	Enrichment of phenotype	Symbols
GOBP	Positively	Integrated stress response signalling	ASNS, ATF3, DDIT3, ATF4, PPP1R15A, HERPUD1
	Positively	Intrinsic apoptotic signalling pathway in response to endoplasmic reticulum stress	TRIB3, CHAC1, DDIT3, ATF4, PDX1, ITPR1, PPP1R15A, MAP3K5, HERPUD1
	Positively	Response to hyperoxia	PDGFRB, CDKN1A, FOXO1, BNIP3, PPARG
	Negatively	Positive regulation of transforming growth factor β production	MYB, SERPINF2, SMAD3, GATA6, FERMT1, CD200, PTGS2
	Negatively	Establishment of mitotic spindle orientation	NDE1, MAD2L1, GPSM2, CENPA, SPDL1, PLK, NDC80
	Negatively	Regulation of lymphocyte chemotaxis	CCL5, PTK2B, ADAM10, CCL2, CXCL14
KEGG	Positively	Glycolysis gluconeogenesis	PCK2, ALDH1B1, ADH1B, ALDOC, ADH1C, ENO2, ADH5, ADH1A, ALDH3A1, HK2, ADH7, PCK1, BPGM, LDHAL6B, ALDOB, ENO3, ALDH2, GPI FBP2, PGK2
	Positively	Histidine metabolism	MAOA, ALDH1B1, ALDH3A1, HNMT
	Positively	Glycine serine and threonine metabolis	PSAT1, MAOA, PHGDH, CBS, AOC3, PSPH, SHMT2, PIPOX, AGXT
	Negatively	Spliceosome	RBM25, DDX46, TRA2A, THOC1, SART1, LSM3, SF3B1, SRSF6, PUF60, THOC2, TXNL4A, SRSF10, SF3B2, SNRPE, PRPF31, TCERG1, SF3B3, PRPF19, SNRPC, PRPF40A, PPIE, SNRPB2, LSM2, DHX8, HNRNPA1, SNRPD1, SRSF3, SNRNP70, U2AF2, DDX23, SNRPD2, SF3B5, TRA2B, LSM6, NCBP1, SRSF7, SNRPA1, PRPF4, SNRPG, DHX15, LSM4, SRSF1, SNRPB, SNRPD3, SNRPF, HNRNPC, LSM7, HNRNPM, SNRPA, PPIH
	Negatively	Parkinson’s disease	PARK7, SDHC, COX4I1, COX6A1, NDUFV2, NDUFB4, COX7A2, NDUFS3, COX7B, COX7C, UBB, UQCRC2, NDUFA10, VDAC1, COX5B, APAF1, NDUFA6, SLC18A2, UBE2J1, COX5A, NDUFB7, UQCRQ, NDUFV, SNCAIP, NDUFA3, COX6B1, UQCR, SDHB, NDUFAB1, NDUFB8, CYC1, NDUFB2, NDUFB1, NDUFS7, SDHA, UBA1, HTRA2, NDUFA1, NDUFA2, NDUFB6, SLC25A5, NDUFS2, NDUFC1, NDUFS1, COX8A, NDUFA7
	Negatively	Proteasome	PSMD11, PSMC5, PSMD6, PSMB4, PSMD8, PSMB9, PSMB7, PSME3, PSMA4, PSMB10, PSMB6, PSMD4, PSMB5, PSMB3, PSMA2, PSMD3, PSMA5, PSMB8, PSMA7, PSMC3, PSMD13, PSMD2, PSME4

#### 3.5.1 BP of GO analysis of all altered genes in GSEA

To better understand the BP of GO of all altered genes during the adipogenesis, BP of GO analysis of all altered genes in GSEA was performed. The top-three significantly enriched gene sets of BP analysis positively correlated with DIF group included integrated stress response signalling, intrinsic apoptotic signalling pathway in response to endoplasmic reticulum stress and response to hyperoxia, meanwhile, the top three of most significant-enriched gene sets positively correlated with the UNDIF group were positive regulation of transforming growth factor β production, establishment of mitotic spindle orientation, and regulation of lymphocyte chemotaxis (Figure 6a, Table 2).

#### 3.5.2 KEGG analysis of all altered genes in GSEA

To better understand the KEGG pathway of all altered genes during the adipogenesis, KEGG analysis of all altered genes in GSEA was performed. The top-three most significantly enriched gene sets in all altered genes of KEGG analysis via GSEA positively correlated with the DIF group, including glycolysis, gluconeogenesis, histidine metabolism, glycine serine and threonine metabolism. Meanwhile, the top-three most significantly enriched gene sets positively correlated with the UNDIF group were spliceosome, Parkinson’s disease, and proteasome (Figure 6b, Table 2).

#### 3.5.3 Hallmark analysis of all altered genes in GSEA

To better understand the hallmark of adipogenesis, hallmark analysis of all altered genes in GSEA was performed. The top-three most significantly enriched gene sets for hallmark analysis in GSEA positively correlated with the DIF group, including unfolded protein response, cholesterol homoeostasis and P53 pathway. The top-three most significant enriched gene sets for hallmark analysis in GSEA positively correlated with the UNDIF group included TGFβ signalling, MYC targets V1, and oxidative phosphorylation (Figure Sa, Table S2).

#### 3.5.4 Oncogenic analysis of all altered genes in GSEA

To declare the oncogene in the adipogenesis, several oncogenic pathways were screened with the performance of oncogenic analysis of all altered genes in GSEA. The top-three most significantly enriched gene sets of oncogenic analysis in GSEA positively correlated with the DIF group included MTOR up.n4.V1 UP, ESC V6.5 UP LATE.V1.DN, and ALK DN.V1 UP. The top-three most significantly enriched gene sets of oncogenic analysis in GSEA positively correlated with the UNDIF group included MYC UP.V1 UP, CSR LATE UP.V1 UP, AND CSR EARLY UP.V1 UP (Figure Sb, Table S2).

## 3.5.5 miRNA analysis of all altered genes in GSEA

To declare the function of miRNAs in the adipogenesis, several miRNAs were screened with the performance of miRNA analysis of all altered genes in GSEA. The top-three most significant enriched gene sets of the altered genes related to miRNA analysis in GSEA positively correlated with the DIF group included miRNA6798-5p, miRNA5684, and miRNA514; The top three of most significant enriched gene sets of miRNA analysis in GSEA positively correlated with the UNDIF group included miRNA4764-3P, miRNA3165, and miRNA6831-3p (Figure Sc, Table S2).

## Discussion

Traditionally, white adipocytes are considered as excess energy storage organs in the form of triglycerides. As an excess energy storage organ, the white adipocytes effectively sequester lipids inside themselves, which prevent other organs such as skeletal muscle, liver from lipotoxicity, staying away from the metabolic dysfunction [19]. However, with the increasing size, the adipocytes become the hypertrophy adipocytes, encountering hypoxia with the limits of oxygen supply, secreting pro-inflammation cytokines, abnormal adipokines, mRNA or noncoding RNA to be implicated in the various metabolism-related disorders, including diabetes, cardiomyopathy and tumours [20–23].

Simultaneously, adipogenesis with the differentiated adipocytes provides more space to store excess lipid with normal size, secreting normal adipokines, mRNA and noncoding RNA to counteract the metabolic disorders. Therefore, adipogenesis of the white adipocytes is emerging as a metabolism mediator on the systemic metabolism homoeostasis [24]. To better understand the contribution of the adipogenesis of the omental white adipocytes to the systemic metabolism homoeostasis, in the current study, bioinformatic methods are performed to analyse the characteristics of the DEGs and the whole altered genes on BP pathways, KEGG pathways, hallmarks, oncogenic pathways, and the related miRNA between the DIF and UNDIF groups.

Based on the mRNA expression data, the 328 DEGs were identified between DIF and UNDIF groups, including 185 upregulated genes and 143 downregulated genes. The BP analysis of GO annotation indicated that the upregulated DEGs were significantly enriched in PERK-mediated unfolded protein response. Previous study demonstrated that PERK played a critical role in the regulation osteoblast differentiation with PERK-EIF2α-ATF4 signalling pathway [25] and in the regulation of myoblast differentiation via miR-128-Ppp1cc network regulated by the PERK signalling pathway [26]. The BP analysis of GO annotation indicated that the downregulated DEGs were significantly enriched in cell proliferation, which was consistent with upregulated DEGs being significantly enriched in negative regulation of cell proliferation.

The KEGG pathway analysis indicated that the upregulated DEGs were significantly enriched in insulin resistance, which included PPP1R3A and PPP1R3C. PPP1R3 is centrally involved in the regulation of lipid metabolism. Previous study indicated that disruption of PPP1R3A promoted the development of insulin resistance. Variant in PPP1R3A is associated with type 2 diabetes [27]. That the expressions of PPP1R3A and PPP1R3C were upregulated in the mature adipocyte, improving the insulin resistance, would give the clue that adipogenesis might be implicated in the insulin sensitivity of white adipocyte.

GSEA is defined as Gene Set Enrichment Analysis, which is used to assess the distribution trend of genes in a predefined Gene Set [18]. In the current study, GSEA analysis differs in several important ways from the altered genes, including GO analysis, KEGG analysis, hallmark analysis, oncogenic analysis and related miRNA analysis.

In the BP of GO analysis of all the altered genes in GSEA, the most significant gene set was enriched in the integrated stress response signalling with the highest enrichment score (ES). The integrated stress response was termed that in response to diverse stress stimuli, eukaryotic cells activate a common adaptive pathway to restore cellular homoeostasis, modulating the ISR to treat ischaemic heart disease, brain ischaemia, ischaemic liver disease, and ischaemic kidney disease. Miyake et al. demonstrated that the integrated stress response played an important role of suppression on the appetite for a high-fat diet and improved obesity via the regulating GDF15 secretion from adipocytes [28]. However, the role of the integrated stress response on the differentiation of the white adipocytes remains elusory. Further study could be carried out on the role of the integrated stress response on the differentiation of the white adipocytes.

In the present study, it was demonstrated that Parkinson’s disease was enriched in UNDIF group with the performance of the KEGG analysis in GSEA. The previous study by Lorefält et al. indicated that fat loss associated with Parkinson’s disease (PD) was positively correlated with the duration of disease [29]. The current study demonstrated that the genes set in the UNDIF were enriched in PD, which would give the hypothesis that the dysfunction of white adipocyte differentiation would be implicated in the onset and process of PD, which might provide a new target for the treatment of PD.

In the hallmark analysis of all altered genes in GSEA, the present study demonstrated that the most significant gene set of DIF group was enriched in unfolded protein response, which was consistent with BP analysis in GO enrichment analysis of DEGs. ASNS, PSAT1 and CHAC1 were the most significant in the gene set of unfolded protein response. Brearley and his colleagues demonstrated that ASNS and PSAT1 might have involved in myogenic differentiation of C2C12 cells [30]. It was illustrated that genetic inhibition of CHAC1 in mice led to lower the muscle mass, demonstrating the importance of CHAC1. However, to date, further study would be carried out on ASNS, PSAT1, and CHAC1 involving the unfolded protein response during the white adipocyte differentiation.

Increasing reports have demonstrated that obesity is associated with the onset and process of the various tumour. In the present study, the oncogenic pathway of the altered genes was illustrated in oncogenic analysis of all altered genes in GSEA. The results demonstrated that in the DIF group, TRIB3 was involved in the first three significant pathway, including MTOR, ESC and ALK pathway. TRIB3 has been reported to be implicated in the breast cancer, lung cancer [31,32]. Interestingly, TRIB3 was involved in the cholesterol homoeostasis pathway, which was one gene set of the hallmark analysis of all altered genes in GSEA. TRIB3 was reported to be upregulated in the adipose tissue of obesity subjects; meanwhile, another report declared that TRIB3 promoted the cholesterol accumulation in the macrophage [33]. Altogether, we could hypothesize that TRIB3 was of great importance during the adipogenesis, which would provide a new target for the treatment of obesity and obesity-related diseases.

MiRNA is a classic noncoding RNA with the length of less than 22 nucleotides, playing a crucial role in various physiological and pathophysiological processes including cell cycle, differentiation, metabolism, and the onset of various diseases. In the present study, miRNA6798-5p, miRNA5684, and miRNA514 were demonstrated to be significantly associated with the genes in the DIF white adipose group. To our knowledge, there were no reports of three miRNAs, neither on the adipogenesis nor on the metabolic disorder.

Although the number of studies on the adipogenesis is increasing, the present study was the first time to analyse the different characteristics between the preadipocytes and the differentiated white adipocytes. The outputs were of great significance for a comprehensive and in-depth understanding of mature adipocyte characteristics. Although the pathways of BP, KEGG, and oncogene, and the altered genes related to miRNA of the differentiated white adipocyte with the bioinformatic analysis were identified, further studies are urgently demanded to study on the effect of the adipogenesis on the systemic metabolism homoeostasis. A greater understanding of what the characteristics of the differentiated white adipocytes could provide novel avenues for the treatment of obesity and obesity-related disorders.

## Supplementary Material

Supplemental Material

## Data Availability

The datasets generated for this study are available on request to the corresponding author.
