# Supplementary material for: Analysis of the different characteristics between omental preadipocytes and differentiated white adipocytes using bioinformatics methods
Source: Adipocyte. 2022 May 1;11(1):227–38. doi: 10.1080/21623945.2022.2063471 (PMC9067510; doi:10.1080/21623945.2022.2063471)
Supplement: Supplemental Material [file KADI_A_2063471_SM4040.zip › supplementary/TableS1.docx]

Table S1: 328 differentially expressed genes (DEGs) between the DIFF and UNDIFF group.

| Gene symbol | Discription | logFC | P.Value | adj.P.Val |
| --- | --- | --- | --- | --- |
| COMP | Cartilage oligomeric matrix protein | 7.402963 | 5.54E-05 | 0.015111 |
| WISP2 | WNT1 inducible signaling pathway protein 2 | 7.011468 | 0.000114 | 0.018172 |
| TMEM176B | Transmembrane protein 176B | 6.221274 | 0.000104 | 0.017069 |
| FABP4 | Fatty acid binding protein 4, adipocyte | 6.178412 | 0.00042 | 0.028582 |
| ADH1B | Alcohol dehydrogenase 1B | 5.502325 | 1.15E-05 | 0.010771 |
| ANGPTL4 | Angiopoietin-like 4 | 5.437698 | 1.01E-06 | 0.00832 |
| PCK1 | Phosphoenolpyruvate carboxykinase 1 (soluble) | 5.317334 | 0.000156 | 0.020778 |
| WFDC1 | WAP four-disulfide core domain 1 | 5.237545 | 3.32E-05 | 0.012874 |
| ASPN | asporin | 5.046491 | 0.000637 | 0.033254 |
| FHL1 | Four and a half LIM domains 1 | 4.628277 | 2.65E-05 | 0.012874 |
| ASNS | Asparagine synthetase (glutamine-hydrolyzing) | 4.35627 | 1.51E-05 | 0.010771 |
| SCN9A | Sodium channel, voltage-gated, type IX, alpha subunit | 4.335718 | 0.000516 | 0.031267 |
| CFD | Complement factor D (adipsin) | 4.141553 | 0.000726 | 0.03614 |
| RGCC | Regulator of cell cycle | 4.046934 | 0.000182 | 0.021661 |
| WNT5B | Wingless-type MMTV integration site family, member 5B | 3.989384 | 0.000468 | 0.030244 |
| GPX3 | Glutathione peroxidase 3 (plasma) | 3.979162 | 1.3E-05 | 0.010771 |
| G0S2 | G0/G1 switch 2 | 3.936087 | 0.00022 | 0.023409 |
| PSAT1 | Phosphoserine aminotransferase 1 | 3.889381 | 4.43E-06 | 0.009268 |
| UPP1 | Uridine phosphorylase 1 | 3.880828 | 8.58E-05 | 0.015707 |
| ELN | Elastin | 3.849057 | 2.52E-06 | 0.00832 |
| CTSK | Cathepsin K | 3.797303 | 3.87E-05 | 0.013297 |
| GPNMB | Glycoprotein (transmembrane) nmb | 3.786421 | 8.65E-05 | 0.015707 |
| CHAC1 | ChaC, cation transport regulator homolog 1 (E. coli) | 3.761634 | 0.000138 | 0.02019 |
| CRLF1 | Cytokine receptor-like factor 1 | 3.692288 | 7.4E-06 | 0.009284 |
| MME | Membrane metallo-endopeptidase | 3.647838 | 0.000654 | 0.033778 |
| CHRDL1 | Chordin-like 1 | 3.625707 | 6.02E-05 | 0.015344 |
| SH3BGR | SH3 domain binding glutamate-rich protein | 3.623686 | 6.7E-06 | 0.009284 |
| LMOD1 | Leiomodin 1 (smooth muscle) | 3.502862 | 8.06E-05 | 0.015707 |
| PAGE4 | P antigen family, member 4 (prostate associated) | 3.473566 | 0.000297 | 0.026275 |
| TRIB3 | Tribbles pseudokinase 3 | 3.449554 | 2.91E-06 | 0.00832 |
| TMEM59L | Transmembrane protein 59-like | 3.291011 | 3.44E-05 | 0.012874 |
| EDNRB | Endothelin receptor type B | 3.238637 | 1.7E-05 | 0.010771 |
| APOE | Apolipoprotein E | 3.208615 | 0.000436 | 0.028982 |
| PCDHGB6 | Protocadherin gamma subfamily B, 6 | 3.194474 | 0.00013 | 0.019884 |
| GEM | GTP binding protein overexpressed in skeletal muscle | 3.176597 | 0.000436 | 0.028982 |
| FST | Follistatin | 3.170998 | 0.000154 | 0.020717 |
| CX3CR1 | Chemokine (C-X3-C motif) receptor 1 | 3.069852 | 0.000364 | 0.027444 |
| PPP1R3C | Protein phosphatase 1, regulatory subunit 3C | 3.045954 | 0.000277 | 0.025655 |
| LRP1B | Low density lipoprotein receptor-related protein 1B | 3.012292 | 0.00035 | 0.026852 |
| FAM135A | Family with sequence similarity 135, member A | 2.995128 | 0.000704 | 0.035486 |
| GPC4 | Glypican 4 | 2.954078 | 0.000288 | 0.02597 |
| C10orf10 | Chromosome 10 open reading frame 10 | 2.919077 | 0.000396 | 0.028582 |
| AOC3 | Amine oxidase, copper containing 3 | 2.908504 | 0.000838 | 0.039405 |
| TMEM176A | Transmembrane protein 176A | 2.798411 | 2.17E-05 | 0.011814 |
| MYO1D | Myosin ID | 2.755349 | 0.000199 | 0.022256 |
| NAG18 | NAG18 mRNA | 2.739362 | 3.98E-05 | 0.013297 |
| NUPR1 | Nuclear protein, transcriptional regulator, 1 | 2.700555 | 1.3E-05 | 0.010771 |
| FOXD1 | Forkhead box D1 | 2.70023 | 0.001251 | 0.048634 |
| GDF15 | Growth differentiation factor 15 | 2.692024 | 9.83E-06 | 0.010771 |
| MEGF6 | Multiple EGF-like-domains 6 | 2.681049 | 0.000334 | 0.026852 |
| MCAM | Melanoma cell adhesion molecule | 2.668984 | 0.001258 | 0.048711 |
| AEBP1 | AE binding protein 1 | 2.668981 | 0.000165 | 0.021126 |
| MAOA | Monoamine oxidase A | 2.666067 | 0.00124 | 0.048625 |
| RRAGD | Ras-related GTP binding D | 2.638238 | 0.000798 | 0.038223 |
| INSM1 | Insulinoma-associated 1 | 2.629567 | 0.000403 | 0.028582 |
| ETV2 | Ets variant 2 | 2.627602 | 3.49E-05 | 0.012874 |
| SRPX | Sushi-repeat containing protein, X-linked | 2.592792 | 1.59E-05 | 0.010771 |
| CSTA | Cystatin A (stefin A) | 2.590847 | 0.0005 | 0.030826 |
| PLA2G16 | Phospholipase A2, group XVI | 2.569805 | 0.00049 | 0.03076 |
| SLC7A11 | Solute carrier family 7 (anionic amino acid transporter light chain, xc- system), member 11 | 2.561373 | 7.9E-05 | 0.015707 |
| ADM | Adrenomedullin | 2.552743 | 0.000141 | 0.020318 |
| PRL | Prolactin | 2.542415 | 0.001218 | 0.048043 |
| ECM2 | Extracellular matrix protein 2, female organ and adipocyte specific | 2.532766 | 0.000529 | 0.031481 |
| LRRC32 | Leucine rich repeat containing 32 | 2.524324 | 0.000253 | 0.024577 |
| SLC6A9 | Solute carrier family 6 (neurotransmitter transporter, glycine), member 9 | 2.52181 | 0.000546 | 0.03173 |
| PDGFRB | Platelet-derived growth factor receptor, beta polypeptide | 2.506459 | 0.000251 | 0.024577 |
| FBLN5 | Fibulin 5 | 2.505422 | 0.001252 | 0.048634 |
| MS4A4A | Membrane-spanning 4-domains, subfamily A, member 4A | 2.499257 | 0.00051 | 0.031224 |
| KCNK3 | Potassium channel, subfamily K, member 3 | 2.473337 | 7.9E-05 | 0.015707 |
| SLC38A1 | Solute carrier family 38, member 1 | 2.430285 | 0.000278 | 0.025655 |
| PLIN2 | perilipin 2 | 2.35269 | 1.8E-05 | 0.010771 |
| COL14A1 | Collagen, type XIV, alpha 1 | 2.293047 | 0.000174 | 0.02123 |
| ATF3 | Activating transcription factor 3 | 2.292078 | 2.97E-05 | 0.012874 |
| RASSF2 | Ras association (RalGDS/AF-6) domain family member 2 | 2.258929 | 0.000153 | 0.020717 |
| CBX7 | Chromobox homolog 7 | 2.239502 | 0.001142 | 0.046065 |
| PRELP | Proline/arginine-rich end leucine-rich repeat protein | 2.237167 | 0.000472 | 0.030288 |
| CAMK2N1 | Calcium/calmodulin-dependent protein kinase II inhibitor 1 | 2.236466 | 0.000122 | 0.019132 |
| FBXO24 | F-box protein 24 | 2.235007 | 0.001081 | 0.045345 |
| NMBR | Neuromedin B receptor | 2.229536 | 0.000501 | 0.030826 |
| CORO2B | Coronin, actin binding protein, 2B | 2.200956 | 0.000222 | 0.023409 |
| CEBPG | CCAAT/enhancer binding protein (C/EBP), gamma | 2.198123 | 0.000174 | 0.02123 |
| ATF5 | Activating transcription factor 5 | 2.196105 | 0.000247 | 0.024577 |
| COL8A1 | Collagen, type VIII, alpha 1 | 2.18431 | 0.000523 | 0.03142 |
| PRUNE2 | Prune homolog 2 (Drosophila) | 2.168181 | 0.000144 | 0.020463 |
| KLF4 | Kruppel-like factor 4 (gut) | 2.158619 | 0.000172 | 0.02123 |
| RAB33A | RAB33A, member RAS oncogene family | 2.158333 | 0.000165 | 0.021126 |
| LILRA1 | Leukocyte immunoglobulin-like receptor, subfamily A (with TM domain), member 1 | 2.118172 | 8.74E-05 | 0.015707 |
| PDE1A | Phosphodiesterase 1A, calmodulin-dependent | 2.111656 | 0.00111 | 0.04581 |
| SPN | Sialophorin | 2.099632 | 0.000231 | 0.023982 |
| S100A1 | S100 calcium binding protein A1 | 2.09535 | 0.000524 | 0.03142 |
| EN1 | engrailed homeobox 1 | 2.092793 | 0.000514 | 0.031267 |
| GADD45G | Growth arrest and DNA-damage-inducible, gamma | 2.078311 | 8.89E-05 | 0.015707 |
| NPAS1 | Neuronal PAS domain protein 1 | 2.074288 | 0.000465 | 0.03023 |
| COL8A2 | Collagen, type VIII, alpha 2 | 2.050292 | 0.000405 | 0.028582 |
| JAG1 | Jagged 1 | 2.038611 | 0.00019 | 0.021819 |
| SPTBN2 | Spectrin, beta, non-erythrocytic 2 | 2.03614 | 0.000676 | 0.034637 |
| KCNK15 | Potassium channel, subfamily K, member 15 | 2.028774 | 0.001121 | 0.045858 |
| PELI2 | Pellino E3 ubiquitin protein ligase family member 2 | 2.028445 | 0.000211 | 0.023054 |
| SNTB1 | Syntrophin, beta 1 (dystrophin-associated protein A1, 59kDa, basic component 1) | 2.016262 | 0.000275 | 0.025655 |
| HIST1H4L | Histone cluster 1, H4l | 2.008251 | 7.02E-05 | 0.015707 |
| SLC1A4 | Solute carrier family 1 (glutamate/neutral amino acid transporter), member 4 | 2.003772 | 4.45E-05 | 0.013297 |
| PHGDH | Phosphoglycerate dehydrogenase | 1.997882 | 0.000295 | 0.026275 |
| CITED2 | Cbp/p300-interacting transactivator, with Glu/Asp-rich carboxy-terminal domain, 2 | 1.962884 | 9.81E-05 | 0.016859 |
| SLC7A2 | Solute carrier family 7 (cationic amino acid transporter, y+ system), member 2 | 1.962831 | 0.000645 | 0.033442 |
| VAC14 | Vac14 homolog (S. cerevisiae) | 1.952078 | 0.000907 | 0.041847 |
| GAS6 | Growth arrest-specific 6 | 1.934889 | 4.1E-05 | 0.013297 |
| ATP10A | ATPase, class V, type 10A | 1.933206 | 0.000316 | 0.026852 |
| JAM2 | Junctional adhesion molecule 2 | 1.929105 | 0.000762 | 0.037262 |
| CCT8L2 | Chaperonin containing TCP1, subunit 8 (theta)-like 2 | 1.928117 | 0.001022 | 0.044228 |
| HOXD11 | Homeobox D11 | 1.919281 | 0.00117 | 0.046754 |
| SCN3A | Sodium channel, voltage-gated, type III, alpha subunit | 1.919124 | 0.000929 | 0.042532 |
| TEX2 | Testis expressed 2 | 1.918507 | 8.57E-05 | 0.015707 |
| PCK2 | Phosphoenolpyruvate carboxykinase 2 (mitochondrial) | 1.903896 | 6.96E-05 | 0.015707 |
| DDIT4 | DNA-damage-inducible transcript 4 | 1.901915 | 0.000334 | 0.026852 |
| TMEM140 | Transmembrane protein 140 | 1.899751 | 0.001049 | 0.044761 |
| TSC22D3 | TSC22 domain family, member 3 | 1.886417 | 5.86E-05 | 0.015344 |
| NEDD9 | Neural precursor cell expressed, developmentally down-regulated 9 | 1.881915 | 0.000339 | 0.026852 |
| PMP22 | Peripheral myelin protein 22 | 1.858519 | 0.000489 | 0.03076 |
| IRS2 | Insulin receptor substrate 2 | 1.842183 | 0.000984 | 0.043803 |
| INSIG2 | Insulin induced gene 2 | 1.821265 | 0.000151 | 0.020717 |
| LITAF | Lipopolysaccharide-induced TNF factor | 1.817266 | 0.000762 | 0.037262 |
| C6orf48 | Chromosome 6 open reading frame 48 | 1.793511 | 0.000164 | 0.021126 |
| MAN1C1 | Mannosidase, alpha, class 1C, member 1 | 1.78903 | 0.000341 | 0.026852 |
| GBE1 | Glucan (1,4-alpha-), branching enzyme 1 | 1.788109 | 5.18E-05 | 0.014442 |
| ADRA2A | Adrenoceptor alpha 2A | 1.784569 | 5.08E-05 | 0.014442 |
| FOXD2 | Forkhead box D2 | 1.77291 | 0.000214 | 0.023127 |
| MTHFD2 | Methylenetetrahydrofolate dehydrogenase (NADP+ dependent) 2, methenyltetrahydrofolate cyclohydrolase | 1.76705 | 6.36E-05 | 0.015344 |
| CLTCL1 | Clathrin, heavy chain-like 1 | 1.758912 | 0.000197 | 0.022245 |
| SLC3A2 | Solute carrier family 3 (amino acid transporter heavy chain), member 2 | 1.746278 | 0.000828 | 0.039052 |
| PRX | Periaxin | 1.742425 | 0.00124 | 0.048625 |
| RPS6KA2 | Ribosomal protein S6 kinase, 90kDa, polypeptide 2 | 1.739891 | 0.000621 | 0.033046 |
| SYTL2 | Synaptotagmin-like 2 | 1.722083 | 0.000367 | 0.027444 |
| LIMS2 | LIM and senescent cell antigen-like domains 2 | 1.721508 | 9.72E-05 | 0.016859 |
| SDC2 | Syndecan 2 | 1.720477 | 0.000694 | 0.035269 |
| EML1 | Echinoderm microtubule associated protein like 1 | 1.716176 | 0.000316 | 0.026852 |
| VLDLR | Very low density lipoprotein receptor | 1.713873 | 0.000169 | 0.02123 |
| DDIT3 | DNA-damage-inducible transcript 3 | 1.697835 | 0.000176 | 0.02123 |
| GSN | gelsolin | 1.693308 | 0.00116 | 0.046489 |
| LMAN1L | Lectin, mannose-binding, 1 like | 1.687288 | 0.000546 | 0.03173 |
| CYBRD1 | Cytochrome b reductase 1 | 1.67855 | 0.001152 | 0.046318 |
| CPQ | Carboxypeptidase Q | 1.676404 | 0.000549 | 0.03173 |
| PPP1R3A | PPP1R3A | 1.665484 | 0.000197 | 0.022245 |
| CCL13 | Chemokine (C-C motif) ligand 13 | 1.644568 | 0.000327 | 0.026852 |
| PTGER4 | Prostaglandin E receptor 4 (subtype EP4) | 1.636898 | 0.000287 | 0.02597 |
| PDX1 | Pancreatic and duodenal homeobox 1 | 1.631162 | 0.001059 | 0.045025 |
| LBR | Lamin B receptor | 1.627717 | 0.000258 | 0.024913 |
| PLEKHF1 | Pleckstrin homology domain containing, family F (with FYVE domain) member 1 | 1.617275 | 0.000353 | 0.026852 |
| ZNF365 | Zinc finger protein 365 | 1.612726 | 0.000281 | 0.025767 |
| BTG1 | B-cell translocation gene 1, anti-proliferative | 1.61098 | 0.00042 | 0.028582 |
| UNC5B | Unc-5 homolog B (C. elegans) | 1.600291 | 0.000452 | 0.029556 |
| CREG1 | Cellular repressor of E1A-stimulated genes 1 | 1.564474 | 0.000413 | 0.028582 |
| JPH2 | Junctophilin 2 | 1.530514 | 0.000236 | 0.024039 |
| ZBTB16 | Zinc finger and BTB domain containing 16 | 1.522362 | 0.000297 | 0.026275 |
| ATF4 | Activating transcription factor 4 | 1.518099 | 0.000146 | 0.020585 |
| SCGN | Secretagogin, EF-hand calcium binding protein | 1.51525 | 0.001128 | 0.045858 |
| SLC31A2 | Solute carrier family 31 (copper transporter), member 2 | 1.478065 | 0.000422 | 0.028582 |
| CDC42EP3 | CDC42 effector protein (Rho GTPase binding) 3 | 1.477159 | 0.000324 | 0.026852 |
| ASS1 | Argininosuccinate synthase 1 | 1.452352 | 0.000335 | 0.026852 |
| TPP1 | Tripeptidyl peptidase I | 1.446924 | 0.000243 | 0.024577 |
| PTPRG | Protein tyrosine phosphatase, receptor type, G | 1.428146 | 0.000211 | 0.023054 |
| ALDH1B1 | Aldehyde dehydrogenase 1 family, member B1 | 1.387865 | 0.00042 | 0.028582 |
| LMO3 | LIM domain only 3 (rhombotin-like 2) | 1.379947 | 0.000612 | 0.033046 |
| VEGFA | Vascular endothelial growth factor A | 1.370184 | 0.000557 | 0.03173 |
| TCEAL4 | Transcription elongation factor A (SII)-like 4 | 1.368711 | 0.000604 | 0.032957 |
| TCEA1 | Transcription elongation factor A (SII), 1 | 1.347934 | 0.000638 | 0.033254 |
| NRCAM | Neuronal cell adhesion molecule | 1.31471 | 0.000567 | 0.03173 |
| ANKH | ANKH inorganic pyrophosphate transport regulator | 1.302888 | 0.000555 | 0.03173 |
| SIAH1 | Siah E3 ubiquitin protein ligase 1 | 1.299307 | 0.000568 | 0.03173 |
| VDR | Vitamin D (1,25- dihydroxyvitamin D3) receptor | 1.274051 | 0.000533 | 0.031576 |
| ASB9 | Ankyrin repeat and SOCS box containing 9 | 1.247877 | 0.000821 | 0.038865 |
| ARHGEF2 | Rho/Rac guanine nucleotide exchange factor (GEF) 2 | 1.246091 | 0.001181 | 0.046895 |
| CPED1 | Cadherin-like and PC-esterase domain containing 1 | 1.243152 | 0.000564 | 0.03173 |
| INHBB | Inhibin, beta B | 1.232194 | 0.001005 | 0.044134 |
| WARS | Tryptophanyl-tRNA synthetase | 1.215107 | 0.000701 | 0.035474 |
| ABCA2 | ATP-binding cassette, sub-family A (ABC1), member 2 | 1.205767 | 0.001278 | 0.049132 |
| SARS | Seryl-tRNA synthetase | 1.182537 | 0.00049 | 0.03076 |
| ACVR1 | Activin A receptor, type I | 1.166765 | 0.000877 | 0.040612 |
| ANK2 | Ankyrin 2, neuronal | 1.147936 | 0.001119 | 0.045858 |
| AIRE | Autoimmune regulator | 1.145321 | 0.000864 | 0.040243 |
| LINC00312 | Long intergenic non-protein coding RNA 312 | 1.141946 | 0.0005 | 0.030826 |
| EPAS1 | Endothelial PAS domain protein 1 | 1.141626 | 0.001194 | 0.047268 |
| LEMD3 | LEM domain containing 3 | 1.105897 | 0.000937 | 0.042586 |
| IARS | Isoleucyl-tRNA synthetase | 1.085219 | 0.000801 | 0.038223 |
| PTTG1IP | Pituitary tumor-transforming 1 interacting protein | 1.051927 | 0.000962 | 0.04326 |
| CDKN1A | Cyclin-dependent kinase inhibitor 1A (p21, Cip1) | 1.005941 | 0.001011 | 0.044134 |
| KRT19 | Keratin 19 | -7.68284 | 0.000302 | 0.026297 |
| CDC20 | Cell division cycle 20 | -5.29721 | 0.000349 | 0.026852 |
| GINS2 | GINS complex subunit 2 (Psf2 homolog) | -5.22301 | 5.97E-05 | 0.015344 |
| ASPM | Asp (abnormal spindle) homolog, microcephaly associated (Drosophila) | -5.09168 | 2.01E-06 | 0.00832 |
| TACC3 | Transforming, acidic coiled-coil containing protein 3 | -4.90872 | 0.000152 | 0.020717 |
| LMNB1 | Lamin B1 | -4.70375 | 0.000249 | 0.024577 |
| CENPM | Centromere protein M | -4.26955 | 2.05E-05 | 0.011711 |
| IL33 | Interleukin 33 | -4.06117 | 1.13E-05 | 0.010771 |
| CXCL6 | Chemokine (C-X-C motif) ligand 6 | -4.06067 | 8.19E-05 | 0.015707 |
| KIAA0101 | KIAA0101 | -3.9912 | 1.57E-05 | 0.010771 |
| PLEK2 | Pleckstrin 2 | -3.94789 | 6.88E-06 | 0.009284 |
| TOP2A | Topoisomerase (DNA) II alpha 170kDa | -3.94227 | 0.000172 | 0.02123 |
| RRM2 | Ribonucleotide reductase M2 | -3.81259 | 0.000189 | 0.021819 |
| NPY | Neuropeptide Y | -3.79684 | 0.001137 | 0.046036 |
| ANXA10 | Annexin A10 | -3.78486 | 0.000135 | 0.020109 |
| KIF18A | kinesin family member 18A | -3.76918 | 0.000043 | 0.013297 |
| LYPD1 | LY6/PLAUR domain containing 1 | -3.74969 | 1.76E-05 | 0.010771 |
| SLPI | Secretory leukocyte peptidase inhibitor | -3.70813 | 0.000639 | 0.033254 |
| PTGS2 | Prostaglandin-endoperoxide synthase 2 (prostaglandin G/H synthase and cyclooxygenase) | -3.68925 | 3.32E-06 | 0.00832 |
| TK1 | Thymidine kinase 1, soluble | -3.62548 | 0.000346 | 0.026852 |
| PAFAH1B3 | Platelet-activating factor acetylhydrolase 1b, catalytic subunit 3 (29kDa) | -3.60177 | 5.78E-06 | 0.009284 |
| DLGAP5 | Discs, large (Drosophila) homolog-associated protein 5 | -3.55184 | 0.000231 | 0.023982 |
| FOXM1 | Forkhead box M1 | -3.50853 | 0.000265 | 0.025387 |
| AADAC | Arylacetamide deacetylase | -3.48982 | 0.000162 | 0.021126 |
| ID1 | Inhibitor of DNA binding 1, dominant negative helix-loop-helix protein | -3.4017 | 0.000136 | 0.020109 |
| STXBP2 | Syntaxin binding protein 2 | -3.36294 | 0.000031 | 0.012874 |
| PRC1 | Peroxisome proliferator-activated receptor gamma, coactivator-related 1 | -3.31143 | 8.72E-05 | 0.015707 |
| CCNB1 | Cyclin B1 | -3.19147 | 0.000132 | 0.019884 |
| CDCA3 | Cell division cycle associated 3 | -3.16213 | 0.00022 | 0.023409 |
| PTTG3P | Pituitary tumor-transforming 3, pseudogene | -3.09926 | 0.000205 | 0.022761 |
| PMEPA1 | Prostate transmembrane protein, androgen induced 1 | -3.07134 | 4.22E-05 | 0.013297 |
| LAS1L | LAS1-like (S. cerevisiae) | -3.05216 | 0.000421 | 0.028582 |
| MCM10 | Minichromosome maintenance complex component 10 | -2.99532 | 0.000553 | 0.03173 |
| PBK | PDZ binding kinase | -2.94921 | 0.000376 | 0.027906 |
| NUSAP1 | Nucleolar and spindle associated protein 1 | -2.94063 | 0.000109 | 0.017487 |
| LRCH1 | Leucine-rich repeats and calponin homology (CH) domain containing 1 | -2.90178 | 2.77E-05 | 0.012874 |
| CFI | Complement factor I | -2.89792 | 0.000604 | 0.032957 |
| LSR | Lipolysis stimulated lipoprotein receptor | -2.87172 | 0.000353 | 0.026852 |
| BIRC5 | Baculoviral IAP repeat containing 5 | -2.86932 | 7.23E-05 | 0.015707 |
| UBE2O | Ubiquitin-conjugating enzyme E2O | -2.86703 | 0.000234 | 0.024039 |
| KIRREL | Kin of IRRE like (Drosophila) | -2.74301 | 0.000105 | 0.017069 |
| CCNB2 | Cyclin B2 | -2.73109 | 4.35E-05 | 0.013297 |
| PACSIN3 | Protein kinase C and casein kinase substrate in neurons 3 | -2.7256 | 3.13E-05 | 0.012874 |
| ID3 | Inhibitor of DNA binding 3, dominant negative helix-loop-helix protein | -2.72346 | 0.001093 | 0.0457 |
| SULT1E1 | Sulfotransferase family 1E, estrogen-preferring, member 1 | -2.69277 | 0.000585 | 0.032215 |
| MXRA5 | Matrix-remodelling associated 5 | -2.6807 | 0.000633 | 0.033254 |
| ENDOG | Endonuclease G | -2.60322 | 0.000624 | 0.033046 |
| GINS4 | GINS complex subunit 4 (Sld5 homolog) | -2.60139 | 7.47E-05 | 0.015707 |
| MMD | Monocyte to macrophage differentiation-associated | -2.59156 | 0.000866 | 0.040243 |
| RAC3 | Ras-related C3 botulinum toxin substrate 3 (rho family, small GTP binding protein Rac3) | -2.5839 | 0.000791 | 0.038058 |
| SLC12A8 | Solute carrier family 12, member 8 | -2.58199 | 0.000578 | 0.032094 |
| GPRC5B | G protein-coupled receptor, class C, group 5, member B | -2.57509 | 0.000341 | 0.026852 |
| PRMT7 | Protein arginine methyltransferase 7 | -2.56815 | 0.000101 | 0.017069 |
| CDK1 | Cyclin-dependent kinase 1 | -2.56781 | 0.000622 | 0.033046 |
| AURKB | Aurora kinase B | -2.55832 | 8.26E-05 | 0.015707 |
| HIST1H4J | Histone cluster 1, H4j | -2.48821 | 3.05E-05 | 0.012874 |
| PYCRL | Pyrroline-5-carboxylate reductase-like | -2.4547 | 0.001126 | 0.045858 |
| RAC2 | Ras-related C3 botulinum toxin substrate 2 (rho family, small GTP binding protein Rac2) | -2.45105 | 0.000954 | 0.043203 |
| GSDMD | Gasdermin D | -2.44007 | 0.000414 | 0.028582 |
| TPX2 | TPX2, microtubule-associated | -2.42251 | 0.000127 | 0.019697 |
| GTF2A1 | General transcription factor IIA, 1, 19/37kDa | -2.40638 | 0.001066 | 0.045025 |
| NRGN | Neurogranin (protein kinase C substrate, RC3) | -2.38472 | 3.12E-05 | 0.012874 |
| CFB | Complement factor B | -2.3823 | 6.31E-05 | 0.015344 |
| CKS2 | CDC28 protein kinase regulatory subunit 2 | -2.37177 | 0.00098 | 0.043778 |
| CXCL14 | Chemokine (C-X-C motif) ligand 14 | -2.36715 | 5.01E-05 | 0.014442 |
| ZGPAT | Zinc finger, CCCH-type with G patch domain | -2.35524 | 0.000034 | 0.012874 |
| KRT5 | Keratin 5 | -2.31648 | 6.24E-05 | 0.015344 |
| NEK2 | NIMA-related kinase 2 | -2.28267 | 0.000716 | 0.035925 |
| FAR2 | Fatty acyl CoA reductase 2 | -2.26606 | 0.000185 | 0.021738 |
| UBE2S | Ubiquitin-conjugating enzyme E2S | -2.23353 | 0.000439 | 0.028982 |
| TYMS | Thymidylate synthetase | -2.23349 | 0.000397 | 0.028582 |
| CHM | Choroideremia (Rab escort protein 1) | -2.23008 | 0.000961 | 0.04326 |
| CENPF | Centromere protein F, 350/400kDa | -2.224 | 8.19E-05 | 0.015707 |
| APOBEC3B | Apolipoprotein B mRNA editing enzyme, catalytic polypeptide-like 3B | -2.20833 | 7.73E-05 | 0.015707 |
| AP1G2 | Adaptor-related protein complex 1, gamma 2 subunit | -2.06624 | 0.000753 | 0.037213 |
| FKBP11 | FK506 binding protein 11, 19 kDa | -2.02218 | 0.000405 | 0.028582 |
| CD200 | CD200 molecule | -1.98291 | 0.000328 | 0.026852 |
| DEPDC1 | DEP domain containing 1 | -1.97861 | 0.000395 | 0.028582 |
| LXN | latexin | -1.97746 | 0.000183 | 0.021661 |
| GCH1 | GTP cyclohydrolase 1 | -1.95758 | 0.000608 | 0.033015 |
| CYP1B1 | Cytochrome P450, family 1, subfamily B, polypeptide 1 | -1.95626 | 0.000277 | 0.025655 |
| PDGFRA | Platelet-derived growth factor receptor, alpha polypeptide | -1.94966 | 0.000367 | 0.027444 |
| SEL1L3 | Sel-1 suppressor of lin-12-like 3 (C. elegans) | -1.94446 | 0.000569 | 0.03173 |
| TEAD1 | TEA domain family member 1 (SV40 transcriptional enhancer factor) | -1.90486 | 0.000322 | 0.026852 |
| BUB1B | BUB1 mitotic checkpoint serine/threonine kinase B | -1.89074 | 0.000501 | 0.030826 |
| STMN1 | Stathmin 1 | -1.88708 | 0.00103 | 0.044425 |
| GPR135 | G protein-coupled receptor 135 | -1.88511 | 3.96E-05 | 0.013297 |
| TRIP13 | Thyroid hormone receptor interactor 13 | -1.87124 | 0.000248 | 0.024577 |
| PLA2G4A | Phospholipase A2, group IVA (cytosolic, calcium-dependent) | -1.83799 | 8.56E-05 | 0.015707 |
| CDKN3 | Cyclin-dependent kinase inhibitor 3 | -1.83022 | 0.001174 | 0.046754 |
| VNN1 | Vanin 1 | -1.8219 | 0.000424 | 0.028582 |
| LDB2 | LIM domain binding 2 | -1.80485 | 0.000561 | 0.03173 |
| HN1 | Hematological and neurological expressed 1 | -1.73965 | 0.000343 | 0.026852 |
| FGF2 | Fibroblast growth factor 2 (basic) | -1.72641 | 0.00111 | 0.04581 |
| WDR46 | WD repeat domain 46 | -1.72139 | 0.001286 | 0.049132 |
| KIF14 | Kinesin family member 14 | -1.68655 | 0.000581 | 0.032115 |
| NFIX | Nuclear factor I/X (CCAAT-binding transcription factor) | -1.68188 | 0.000443 | 0.0291 |
| TFPI2 | Tssue factor pathway inhibitor 2 | -1.68178 | 0.000274 | 0.025655 |
| UGCG | UDP-glucose ceramide glucosyltransferase | -1.67387 | 8.87E-05 | 0.015707 |
| MAPRE2 | Microtubule-associated protein, RP/EB family, member 2 | -1.65884 | 0.000747 | 0.037029 |
| CYBA | Cytochrome b-245, alpha polypeptide | -1.64793 | 0.0003 | 0.026291 |
| NFAT5 | Nuclear factor of activated T-cells 5, tonicity-responsive | -1.63491 | 0.000792 | 0.038058 |
| GFPT2 | Glutamine-fructose-6-phosphate transaminase 2 | -1.60965 | 0.001246 | 0.048634 |
| DHFR | dihydrofolate reductase | -1.58898 | 0.001022 | 0.044228 |
| CDH6 | Cadherin 6, type 2, K-cadherin (fetal kidney) | -1.58597 | 0.000103 | 0.017069 |
| NCAPG2 | Non-SMC condensin II complex, subunit G2 | -1.58551 | 0.000788 | 0.038058 |
| PNP | Purine nucleoside phosphorylase | -1.55336 | 0.000526 | 0.03142 |
| BUB1 | BUB1 mitotic checkpoint serine/threonine kinase | -1.55189 | 0.001005 | 0.044134 |
| WASL | Wiskott-Aldrich syndrome-like | -1.5437 | 0.000473 | 0.030288 |
| PEG10 | Paternally expressed 10 | -1.5188 | 0.000856 | 0.040058 |
| LMNB2 | Lamin B2 | -1.51662 | 0.00104 | 0.044581 |
| KCNK1 | Potassium channel, subfamily K, member 1 | -1.50593 | 0.000975 | 0.043675 |
| SULT1A2 | Sulfotransferase family, cytosolic, 1A, phenol-preferring, member 2 | -1.49545 | 0.000616 | 0.033046 |
| TEAD4 | TEA domain family member 4 | -1.47243 | 0.000439 | 0.028982 |
| HDAC1 | Histone deacetylase 1 | -1.47165 | 0.000781 | 0.038009 |
| SMAD5 | SMAD family member 5 | -1.43663 | 0.000353 | 0.026852 |
| MYO10 | Myosin X | -1.40139 | 0.000684 | 0.034869 |
| CCDC88A | Coiled-coil domain containing 88A | -1.39715 | 0.00056 | 0.03173 |
| PRRC1 | Proline-rich coiled-coil 1 | -1.39523 | 0.001108 | 0.04581 |
| USP10 | Ubiquitin specific peptidase 10 | -1.38797 | 0.000408 | 0.028582 |
| TTC38 | Tetratricopeptide repeat domain 38 | -1.38377 | 0.000419 | 0.028582 |
| PPIH | Peptidylprolyl isomerase H (cyclophilin H) | -1.3689 | 0.000422 | 0.028582 |
| BRF1 | BRF1, RNA polymerase III transcription initiation factor 90 kDa subunit | -1.36737 | 0.001108 | 0.04581 |
| PDP1 | Pyruvate dehyrogenase phosphatase catalytic subunit 1 | -1.36537 | 0.001041 | 0.044581 |
| NDUFA7 | NADH dehydrogenase (ubiquinone) 1 alpha subcomplex, 7, 14.5kDa | -1.3543 | 0.000484 | 0.03076 |
| DPP4 | Dipeptidyl-peptidase 4 | -1.349 | 0.001129 | 0.045858 |
| NME3 | NME/NM23 nucleoside diphosphate kinase 3 | -1.34578 | 0.000616 | 0.033046 |
| GGH | Gamma-glutamyl hydrolase (conjugase, folylpolygammaglutamyl hydrolase) | -1.31861 | 0.00092 | 0.042264 |
| HRASLS | HRAS-like suppressor | -1.31579 | 0.001013 | 0.044134 |
| WHSC1 | Wolf-Hirschhorn syndrome candidate 1 | -1.30406 | 0.000816 | 0.038784 |
| MLEC | Malectin | -1.30274 | 0.000315 | 0.026852 |
| SEC23IP | SEC23 interacting protein | -1.30261 | 0.000763 | 0.037262 |
| ZNF280B | Zinc finger protein 280B | -1.30167 | 0.000564 | 0.03173 |
| AP1S1 | Adaptor-related protein complex 1, sigma 1 subunit | -1.28715 | 0.001006 | 0.044134 |
| ARPC1B | Actin related protein 2/3 complex, subunit 1B, 41kDa | -1.26007 | 0.00066 | 0.033956 |
| HS2ST1 | Heparan sulfate 2-O-sulfotransferase 1 | -1.19104 | 0.000935 | 0.042586 |
| HMOX2 | heme oxygenase (decycling) 2 | -1.17734 | 0.001288 | 0.049132 |
| TRNAU1AP | tRNA selenocysteine 1 associated protein 1 | -1.12112 | 0.00072 | 0.036009 |
| SEPW1 | Selenoprotein W, 1 | -1.07819 | 0.001065 | 0.045025 |
| KDM1A | Lysine (K)-specific demethylase 1A | -1.07762 | 0.001285 | 0.049132 |
| CDC42EP2 | CDC42 effector protein (Rho GTPase binding) 2 | -1.02133 | 0.001013 | 0.044134 |
| SYNJ2 | Synaptojanin 2 | -1.01682 | 0.001267 | 0.048904 |
| EDC4 | Enhancer of mRNA decapping 4 | -1.01584 | 0.00108 | 0.045345 |
