# Supplementary material for: Analysis of the different characteristics between omental preadipocytes and differentiated white adipocytes using bioinformatics methods
Source: Adipocyte. 2022 May 1;11(1):227–38. doi: 10.1080/21623945.2022.2063471 (PMC9067510; doi:10.1080/21623945.2022.2063471)
Supplement: Supplemental Material [file KADI_A_2063471_SM4040.zip › supplementary/TableS2.docx]

Table 2 Gene sets enrichment in GSEA analysis on

| **Type of GSEA** | **Correlated with the differentiated white adipocytes** | **Enrichment of phenotype** | **Symbols** |
| --- | --- | --- | --- |
| Hallmark analysis | Positively | Unfolded protein response | ASNS, PSAT1, CHAC1, ATF3, SLC1A4, MTHFD2, ATF4, VEGFA, CEBPG, SLC7A5, DDIT4, XPOT, HERPUD1, EXOSC1, DNAJB9, WFS1C, SDAD1, CHSPA9 |
|  | Positively | Cholesterol homeostasis, and | TRIB3, ATF3, JAG1, ABCA2, ATF5, NFIL3, CBS, ALDOC, SREBF2, PPARG, SCD, LDLR, STX5, LGALS3, SC5D, LPL, FASN, PCYT2, PDK3, ECH1, GNAI1HMGCR, HSD17B7, PNRC1, SQLE, FDFT1, ANXA13, IDI1, TM7SF2, AVPR1A, DHCR7 |
|  | Positively | P53 pathway | TRIB3, UPP1, NUPR1, ATF3, KLF4, DDIT3, ZBTB16, BTG1, SLC7A11, ZNF365, SLC3A2, VDR, DDIT4, INHBB, SLC19A2, ABHD4, PPP1R15A, CDKN1A, SP1, ZMAT3, GADD45A, TXNIP, F2R, STOM, TCN2, CDH13, CEBPA, AEN, CTSD, KLK8, JAG2, TSC22D1, TCHH, PRKAB1, FOXO3, EPHX1, MKNK2, ISCU, VWA5A, SERPINB5, HMOX1, RAD9A, SDC1, RETSAT, POLH, TGFA, GPX2, ALOX15B, OSGIN1, NDRG1, CDKN2A, BTG2, CGRRF1, KIF13B, DRAM1 |
|  | Negatively | TGFβ signaling， | [CDH1](https://ensembl.org/Search/Results?q=CDH1), [SMURF2](https://ensembl.org/Search/Results?q=SMURF2), [SMAD3](https://ensembl.org/Search/Results?q=SMAD3), [SPTBN1](https://ensembl.org/Search/Results?q=SPTBN1), [BMP2](https://ensembl.org/Search/Results?q=BMP2), [SMAD6](https://ensembl.org/Search/Results?q=SMAD6), [BMPR2](https://ensembl.org/Search/Results?q=BMPR2), [SLC20A1](https://ensembl.org/Search/Results?q=SLC20A1), [PPP1CA](https://ensembl.org/Search/Results?q=PPP1CA), [ID1](https://ensembl.org/Search/Results?q=ID1), [ID3](https://ensembl.org/Search/Results?q=ID3), [HDAC1](https://ensembl.org/Search/Results?q=HDAC1), [PMEPA1](https://ensembl.org/Search/Results?q=PMEPA1) |
|  | Negatively | MYC targets V1 | [SSB](https://ensembl.org/Search/Results?q=SSB), [DDX18](https://ensembl.org/Search/Results?q=DDX18) [ACP1](https://ensembl.org/Search/Results?q=ACP1), [TRIM28](https://ensembl.org/Search/Results?q=TRIM28), [PHB2](https://ensembl.org/Search/Results?q=PHB2), [TXNL4A](https://ensembl.org/Search/Results?q=TXNL4A), [COX5A](https://ensembl.org/Search/Results?q=COX5A), [YWHAQ](https://ensembl.org/Search/Results?q=YWHAQ), [PSMA4](https://ensembl.org/Search/Results?q=PSMA4), [PPIA](https://ensembl.org/Search/Results?q=PPIA), [CDK2](https://ensembl.org/Search/Results?q=CDK2), [PRPF31](https://ensembl.org/Search/Results?q=PRPF31), [SF3B3](https://ensembl.org/Search/Results?q=SF3B3), [EIF1AX](https://ensembl.org/Search/Results?q=EIF1AX), [SLC25A3](https://ensembl.org/Search/Results?q=SLC25A3), [POLD2](https://ensembl.org/Search/Results?q=POLD2), [PSMB3](https://ensembl.org/Search/Results?q=PSMB3), [IMPDH2](https://ensembl.org/Search/Results?q=IMPDH2), [EXOSC7](https://ensembl.org/Search/Results?q=EXOSC7), [SNRPB2](https://ensembl.org/Search/Results?q=SNRPB2), [PABPC1](https://ensembl.org/Search/Results?q=PABPC1), [RAN](https://ensembl.org/Search/Results?q=RAN), [NDUFAB1](https://ensembl.org/Search/Results?q=NDUFAB1), [RRP9](https://ensembl.org/Search/Results?q=RRP9), [HNRNPR](https://ensembl.org/Search/Results?q=HNRNPR), [CYC1](https://ensembl.org/Search/Results?q=CYC1), [ODC1](https://ensembl.org/Search/Results?q=ODC1), [HDAC2](https://ensembl.org/Search/Results?q=HDAC2), [HPRT1](https://ensembl.org/Search/Results?q=HPRT1), [SRPK1](https://ensembl.org/Search/Results?q=SRPK1), [TFDP1](https://ensembl.org/Search/Results?q=TFDP1), [FBL](https://ensembl.org/Search/Results?q=FBL), [DDX21](https://ensembl.org/Search/Results?q=DDX21), [NOP16](https://ensembl.org/Search/Results?q=NOP16), [BUB3](https://ensembl.org/Search/Results?q=BUB3), [APEX1](https://ensembl.org/Search/Results?q=APEX1), [PWP1](https://ensembl.org/Search/Results?q=PWP1), [TUFM](https://ensembl.org/Search/Results?q=TUFM), [GSPT1](https://ensembl.org/Search/Results?q=GSPT1), [CAD](https://ensembl.org/Search/Results?q=CAD), [LSM2](https://ensembl.org/Search/Results?q=LSM2), [XRCC6](https://ensembl.org/Search/Results?q=XRCC6), [HNRNPA2B1](https://ensembl.org/Search/Results?q=HNRNPA2B1), [NPM1](https://ensembl.org/Search/Results?q=NPM1), [GOT2](https://ensembl.org/Search/Results?q=GOT2), [EIF2S1](https://ensembl.org/Search/Results?q=EIF2S1), [FAM120A](https://ensembl.org/Search/Results?q=FAM120A), [CCT5](https://ensembl.org/Search/Results?q=CCT5), [HNRNPA1](https://ensembl.org/Search/Results?q=HNRNPA1), [SNRPD1](https://ensembl.org/Search/Results?q=SNRPD1), [SRSF3](https://ensembl.org/Search/Results?q=SRSF3), [SERBP1](https://ensembl.org/Search/Results?q=SERBP1), [MYC](https://ensembl.org/Search/Results?q=MYC), [SMARCC1](https://ensembl.org/Search/Results?q=SMARCC1), [CDC45](https://ensembl.org/Search/Results?q=CDC45), [PRPS2](https://ensembl.org/Search/Results?q=PRPS2), [SNRPD2](https://ensembl.org/Search/Results?q=SNRPD2), [PA2G4](https://ensembl.org/Search/Results?q=PA2G4), [TRA2B](https://ensembl.org/Search/Results?q=TRA2B), [KPNA2](https://ensembl.org/Search/Results?q=KPNA2), [NCBP1](https://ensembl.org/Search/Results?q=NCBP1), [MCM2](https://ensembl.org/Search/Results?q=MCM2), [SRSF7](https://ensembl.org/Search/Results?q=SRSF7), [NOLC1](https://ensembl.org/Search/Results?q=NOLC1), [TARDBP](https://ensembl.org/Search/Results?q=TARDBP), [SNRPA1](https://ensembl.org/Search/Results?q=SNRPA1), [MCM4](https://ensembl.org/Search/Results?q=MCM4), [EIF3B](https://ensembl.org/Search/Results?q=EIF3B), [ILF2](https://ensembl.org/Search/Results?q=ILF2), [SYNCRIP](https://ensembl.org/Search/Results?q=SYNCRIP), [PSMA2](https://ensembl.org/Search/Results?q=PSMA2), [HSP90AB1](https://ensembl.org/Search/Results?q=HSP90AB1), [PSMD3](https://ensembl.org/Search/Results?q=PSMD3), [MRPL23](https://ensembl.org/Search/Results?q=MRPL23), [CCT2](https://ensembl.org/Search/Results?q=CCT2), [SNRPG](https://ensembl.org/Search/Results?q=SNRPG), [CSTF2](https://ensembl.org/Search/Results?q=CSTF2), [CCT4](https://ensembl.org/Search/Results?q=CCT4), [HNRNPD](https://ensembl.org/Search/Results?q=HNRNPD), [HSPE1](https://ensembl.org/Search/Results?q=HSPE1), [DHX15](https://ensembl.org/Search/Results?q=DHX15), [PSMA7](https://ensembl.org/Search/Results?q=PSMA7), [MAD2L1](https://ensembl.org/Search/Results?q=MAD2L1), [SRM](https://ensembl.org/Search/Results?q=SRM), [SRSF1](https://ensembl.org/Search/Results?q=SRSF1), [C1QBP](https://ensembl.org/Search/Results?q=C1QBP), [SNRPD3](https://ensembl.org/Search/Results?q=SNRPD3), [HSPD1](https://ensembl.org/Search/Results?q=HSPD1), [PHB](https://ensembl.org/Search/Results?q=PHB), [CCNA2](https://ensembl.org/Search/Results?q=CCNA2), [SET](https://ensembl.org/Search/Results?q=SET), [HNRNPC](https://ensembl.org/Search/Results?q=HNRNPC), [CCT7](https://ensembl.org/Search/Results?q=CCT7), [RRM1](https://ensembl.org/Search/Results?q=RRM1), [LSM7](https://ensembl.org/Search/Results?q=LSM7), [SNRPA](https://ensembl.org/Search/Results?q=SNRPA), [DUT](https://ensembl.org/Search/Results?q=DUT), [MCM5](https://ensembl.org/Search/Results?q=MCM5), [NME1](https://ensembl.org/Search/Results?q=NME1), [RUVBL2](https://ensembl.org/Search/Results?q=RUVBL2), [KPNB1](https://ensembl.org/Search/Results?q=KPNB1), [MCM7](https://ensembl.org/Search/Results?q=MCM7), [NHP2](https://ensembl.org/Search/Results?q=NHP2), [TYMS](https://ensembl.org/Search/Results?q=TYMS), [RANBP1](https://ensembl.org/Search/Results?q=RANBP1), [CDC20](https://ensembl.org/Search/Results?q=CDC20) |
|  | Negatively | Oxidative phosphorylation | [IDH3B](https://ensembl.org/Search/Results?q=IDH3B), [SDHC](https://ensembl.org/Search/Results?q=SDHC), [COX4I1](https://ensembl.org/Search/Results?q=COX4I1), [COX6A1](https://ensembl.org/Search/Results?q=COX6A1), [ETFA](https://ensembl.org/Search/Results?q=ETFA), [SLC25A11](https://ensembl.org/Search/Results?q=SLC25A11), [NDUFV2](https://ensembl.org/Search/Results?q=NDUFV2), [NDUFB4](https://ensembl.org/Search/Results?q=NDUFB4), [TIMM8B](https://ensembl.org/Search/Results?q=TIMM8B), [COX7A2](https://ensembl.org/Search/Results?q=COX7A2), [TOMM22](https://ensembl.org/Search/Results?q=TOMM22), [NDUFS3](https://ensembl.org/Search/Results?q=NDUFS3), [COX7B](https://ensembl.org/Search/Results?q=COX7B), [TIMM10](https://ensembl.org/Search/Results?q=TIMM10), [BDH2](https://ensembl.org/Search/Results?q=BDH2), [MFN2](https://ensembl.org/Search/Results?q=MFN2), [COX7C](https://ensembl.org/Search/Results?q=COX7C), [UQCRC2](https://ensembl.org/Search/Results?q=UQCRC2), [PDHA1](https://ensembl.org/Search/Results?q=PDHA1), [ECHS1](https://ensembl.org/Search/Results?q=ECHS1), [TCIRG1](https://ensembl.org/Search/Results?q=TCIRG1), [OAT](https://ensembl.org/Search/Results?q=OAT), [VDAC1](https://ensembl.org/Search/Results?q=VDAC1), [COX5B](https://ensembl.org/Search/Results?q=COX5B), [LDHB](https://ensembl.org/Search/Results?q=LDHB), [NDUFA6](https://ensembl.org/Search/Results?q=NDUFA6) [CS](https://ensembl.org/Search/Results?q=CS), [ACAA1](https://ensembl.org/Search/Results?q=ACAA1) [PDHB](https://ensembl.org/Search/Results?q=PDHB), [ATP6V0B](https://ensembl.org/Search/Results?q=ATP6V0B), [PHB2](https://ensembl.org/Search/Results?q=PHB2), [COX5A](https://ensembl.org/Search/Results?q=COX5A), [MTRR](https://ensembl.org/Search/Results?q=MTRR), [NDUFB7](https://ensembl.org/Search/Results?q=NDUFB7), [POLR2F](https://ensembl.org/Search/Results?q=POLR2F), [UQCRQ](https://ensembl.org/Search/Results?q=UQCRQ), [NDUFV1](https://ensembl.org/Search/Results?q=NDUFV1), [FXN](https://ensembl.org/Search/Results?q=FXN), [NDUFA3](https://ensembl.org/Search/Results?q=NDUFA3), [COX6B1](https://ensembl.org/Search/Results?q=COX6B1), [SLC25A3](https://ensembl.org/Search/Results?q=SLC25A3), [IDH2](https://ensembl.org/Search/Results?q=IDH2), [UQCR10](https://ensembl.org/Search/Results?q=UQCR10), [SDHB](https://ensembl.org/Search/Results?q=SDHB), [NDUFAB1](https://ensembl.org/Search/Results?q=NDUFAB1), [NDUFB8](https://ensembl.org/Search/Results?q=NDUFB8), [CYC1](https://ensembl.org/Search/Results?q=CYC1), [MDH2](https://ensembl.org/Search/Results?q=MDH2), [NDUFB2](https://ensembl.org/Search/Results?q=NDUFB2), [NDUFB1](https://ensembl.org/Search/Results?q=NDUFB1), [MDH1](https://ensembl.org/Search/Results?q=MDH1), [IDH3A](https://ensembl.org/Search/Results?q=IDH3A), [NDUFS7](https://ensembl.org/Search/Results?q=NDUFS7), [SDHA](https://ensembl.org/Search/Results?q=SDHA), [ETFB](https://ensembl.org/Search/Results?q=ETFB), [GOT2](https://ensembl.org/Search/Results?q=GOT2), [IDH3G](https://ensembl.org/Search/Results?q=IDH3G), [HTRA2](https://ensembl.org/Search/Results?q=HTRA2), [RHOT2](https://ensembl.org/Search/Results?q=RHOT2), [NDUFA1](https://ensembl.org/Search/Results?q=NDUFA1), [BAX](https://ensembl.org/Search/Results?q=BAX), [NDUFA2](https://ensembl.org/Search/Results?q=NDUFA2), [MRPS15](https://ensembl.org/Search/Results?q=MRPS15), [NDUFB6](https://ensembl.org/Search/Results?q=NDUFB6), [ABCB7](https://ensembl.org/Search/Results?q=ABCB7), [PMPCA](https://ensembl.org/Search/Results?q=PMPCA), [FH](https://ensembl.org/Search/Results?q=FH), [SLC25A5](https://ensembl.org/Search/Results?q=SLC25A5), [NDUFS2](https://ensembl.org/Search/Results?q=NDUFS2), [NDUFC1](https://ensembl.org/Search/Results?q=NDUFC1), [NDUFS1](https://ensembl.org/Search/Results?q=NDUFS1), [MRPL35](https://ensembl.org/Search/Results?q=MRPL35), [SUCLG1](https://ensembl.org/Search/Results?q=SUCLG1), [COX8A](https://ensembl.org/Search/Results?q=COX8A), [TIMM13](https://ensembl.org/Search/Results?q=TIMM13), [MRPS12](https://ensembl.org/Search/Results?q=MRPS12), [HSD17B10](https://ensembl.org/Search/Results?q=HSD17B10), [NDUFA7](https://ensembl.org/Search/Results?q=NDUFA7), [PDP1](https://ensembl.org/Search/Results?q=PDP1) |
| Oncogenic analysis | Positively | MTOR up.n4.V1 UP | ASNS, TRIB3, CHAC1, ATF3, PCK2, TSC22D3, GBE1, VLDLR, ADM, DDIT3, PHGDH, ASS1, SLC7A11, VEGFA, SLC6A9, CEBPG, SLC3A2, SLC7A5, DDIT4, NFIL3, ITPR1, OPN3, TBRG4, ULBP, HERPUD1, MFAP4, PIR, ABHD6, BNIP3, TIMM44, LHX2, MSMO1, SLC31A1, RIT1, LDLR, RIMS3, PCCA, IFRD1, PSPH, APOL6 |
|  | Positively | ESC V6.5 UP LATE.V1.DN | TRIB3, NUPR1, CHAC1, ANGPTL4, KLF4, MGP, VAC14, CBX7, INHBB, ABCA2, JAM2, PRX, FOXD3, GADD45A, EXOSC1, NEFL, PDGFC, GJB3, RASGRP2, DDX4, AVPI1, TFEB, GPATCH4, LGALS3, SPP1, GPX2, ACACB, HADHB, PCOLCE2 |
|  | Positively | ALK DN.V1 UP | ASNS, TRIB3, PSAT1, GDF15 , CHAC1, ANGPTL4, PCK2, MTHFD2, SLC7A11, VEGFA, SLC6A9, CEBPG, INHBE, SLC7A5, DDIT4, EPAS1, B3GALT4 , CBS, CSTA, GRM1, FOXP3, CTRB2, DLX6, ENTPD3 |
|  | Negatively | MYC UP.V1 UP | PIM2, SORD, MPHOSPH10, AGPAT3, TFB2M, UBIAD1, UTP14A, RRP12, NOP14, CGREF1, TAF5, POLR1E, TSFM, SLC25A22, CALML4, S100A10, RRP9, PUS7, CHKA, PUS1, RPP40, SLC25A15, GLRX5, ZNF667, C20orf27, EEF1E1, EXOSC5, DCTPP1, PRR7, TRMT1, ZNF593, NOL6, IPO4, HSPE1, FAM216A, ISOC2, QTRT1, P2RX5, HS3ST3A1, AMPD2, ZNF215, PMM2, MRTO4, NEFH, LAS1L, PES1, SAC3D1, AHSA1, TRNAU1AP, DENND2, PLA2G4A |
|  | Negatively | CSR LATE UP.V1 UP, AND | CIAPIN1, POLR3K, POLE3, TIMP1, FARSA, NOC2L, KCNC4, MRAS, KISS1, CDC6, TUBG1, S1PR1, IL7R, HERC4, NUDC, EIF5A, GREM2, VEGFC, UTP18, ITGA6, CDC7, TOMM34, IMP4, ASB6, POLD1, FANCA, BEX1, GMNN, NDC1, EEF1E1, GINS3, CDCA8, SMS, PA2G4, DCTPP1, NSG1, MTHFD1, MCM3, DTYMK, FEN1, SNRPA1, EIF3B, ILF2, KIF22, DRAP1, UBE2C, FAM216A, PAICS , PSMC3, SRM, CKAP2, HMGB1, MYBL2, PSMD2, CDK1, ZWINT, PRMT5, CDK5, CCND3, NETO2, KRT18, SHCBP1, AHSA1, RRM2, LMNB2, PBK, ZWILCH, CXCL6, TSPAN13, BIRC5, FOXM1 |
|  | Negatively | CSR EARLY UP.V1 UP | SMARCA4, UBE2N, PTPN1, UAP1, RRP9, KLF13, ZPBP, DDX21, REST, NOP16, SHB, SRP72, C21orf91, ST3GAL1, PLAUR, NCL, NET1, PPIF, UTP3, NSG1, IL11, ILF2, PCSK7, BDKRB1 , EHD4, NRIP1, AMD1, TOP1, NT5E, ID3, PNP, PTGS2 |
| MiRNA analysis | Positively | miRNA6798-5p | NEDD9, MGP, PCYT1B, SERPINA1, EFNA3, COL10A1, FN3K, SYK, FAM193A, PAX5, ATP6AP2 |
|  | Positively | miRNA5684 | LILRA1, SNTB1, FST, RCOR3, CBX8 |
|  | Positively | miRNA514 | PTPRG, NRXN3, SCN3A, SVIL, VAT1, AR, SYT11 , TAL1, PCCA, C7, ZIC1 , AGAP1, TCF12, BAALC, AFF4, NR3C1, EIF4ENIF1, FOXO4 |
|  | Negatively | miRNA4764-3P, | POU2F1, MAP3K1, SNRPD1, RFWD3, TBC1D1, LAMB1, MAD2L1, KPNB1, ATXN7L1, USP10, CYP1B1 |
|  | Negatively | miRNA3165 | ENTPD6, NEK4, ACIN1, PPARA, SCN1B, PURA, ARMCX2, RFX5, DERL2, CAPZA1, HDAC2, SRPK1, PHB , SYNJ2, NCAPG2 |
|  | Negatively | miRNA6831-3p | COPS2, BICD2, GRIA2, MTF2, BTC, PIN4, APAF1, SYNC, SLC22A4, CNOT7, ETNK1, ABAT, IL1A, PRKG1, CD200 |
